# Tailoring InSb Nanowires for High Thermoelectric Performance Using AAO Template-Assisted Die Casting Process

**DOI:** 10.3390/nano12122032

**Published:** 2022-06-13

**Authors:** Alangadu Kothandan Vivekanandan, Chen-Wei Lee, Rui-Zhe Wu, Wei-Han Tsai, Shih-Hsun Chen, Yang-Yuan Chen, Chia-Ting Lin

**Affiliations:** 1 Department of Mechanical Engineering, National Taiwan University of Science and Technology, Taipei 10607, Taiwan; vivekak77@gmail.com (A.K.V.); chenweibear1007@gmail.com (C.-W.L.); jerrywu4416@gmail.com (R.-Z.W.); 2 Institute of Physics, Academia Sinica, Taipei 11529, Taiwan; ham0444@gmail.com; 3 Chemical Systems Research Division, National Chung-Shan Institute of Science and Technology, Taoyuan 325, Taiwan; w409635@gmail.com

**Keywords:** InSb, nanowire, vacuum die-casting, anodic aluminium oxide, thermoelectric properties

## Abstract

Herein, we demonstrate a facile technique for the fabrication of one-dimensional indium antimonide (InSb) nanowires using anodic aluminium oxide (AAO) template-assisted vacuum die-casting method. The filling mechanism of the vacuum die-casting process is investigated on varying AAO pore structures through different electrolytes. It is found that the anodizing electrolytes play a vital role in nanowire growth and structure formation. The as-obtained InSb nanowires from the dissolution process show a degree of high crystallinity, homogeneity, and uniformity throughout their structure. The TEM and XRD results elucidated the InSb zinc-blende crystal structure and preferential orientation along the c-axis direction. The thermoelectric characteristics of InSb nanowires were measured with a four-electrode system, and their resistivity, Seebeck coefficient, power factor, thermal conductivity, and ZT have been evaluated. Further, surface-modified nanowires using the reactive-ion etching technique showed a 50% increase in thermoelectric performance.

## 1. Introduction

The existing conventional energy systems unavoidably face severe consequences of increasing cost, use of fossil fuels, lower efficiencies, and wasted heat. The apparent barriers demand an alternative strategy to replace several ineffective traditional approaches [1,2,3,4]. In recent years, thermoelectric technology has emerged as a promising candidate to meet global requirements in the field of new energy systems [5,6]. The direct energy conversion of thermoelectric materials between heat and electricity elucidates the predominant advantage of utilizing waste heat from environmental and industrial processes. The energy conversion efficiency of thermoelectric materials is determined by the Seebeck coefficient (S), total thermal conductivity (κ), and electrical conductivity (σ). The dimensionless figure of merit ZT (ZT = S^2^σT/κ) has been defined to quantify thermoelectric performance [7]. The functional ZT is constrained mainly by thermoelectric material nanostructure, electronic band structure, thermal conductivity minimization aspects, and high Seebeck values of materials [8,9]. Indeed, the theoretical and experimental studies have emphasized the importance of employing one-dimensional nanostructured materials, especially nanowires (NWs), to increase the ZT value, which has the dual effect of lowering thermal conductivity and improving the Seebeck value. The surface-to-volume ratio and quantum confinement effect can strongly influence the thermoelectric characteristics of the NWs. Alongside this, some underlying mechanisms such as kink morphology, acoustic softening, and surface roughness play a role in reducing the thermal conductivity of NWs, which helps attain enhanced ZT [2,10,11]. However, attaining precise, high-quality, uniform NWs on a large scale is still a significant concern, which offers a rich opportunity to explore NW synthesis in diverse material systems.

Tailoring the NW geometry with high homogeneity is the key challenge in bottom-up and top-down synthesis processes. Several hybrid methods were developed to provide merits and unique attributes in the synthesis of precisely controlled NWs. For instance, the art of pre-patterning the substrate in the bottom-up approach along with the vapor–liquid–solid growth process yields a well-defined ordered array of vertically aligned NWs [12,13]. However, the commonly adapted NW synthesis processes are discharge [14], cation ex-change [15], chemical vapor deposition [16], electrodeposition [17], laser ablation [18], selected-control deposition [16], the sol-gel method [19], thermal decomposition [20], and template methods [21,22]. Among these techniques, the template-assisted method, particularly porous anodic aluminum oxide (AAO, i.e., Al_2_O_3_), has been extensively reported due to its simple fabrication process and low cost [21]. AAO consists of a regularly ordered, vertically oriented honeycomb-like structure, ideal for explicit nanomold use. The cylindrical pore section in the AAO can be regulated from the sub-micro to nanoscale range [23,24]. In AAO, the growth and hemispherical geometry of the AAO channels largely depends on the anodizing parameters (electrolyte, anodic voltage, treatment temperature, and time) [25]. The regularities of nanochannel, pore diameter, and pore density of AAO are predominately controlled by anodizing electrolytes such as oxalic, phosphoric, and sulfuric acid [26]. The fabricated highly ordered AAO templates are deployed as nano-structuration/nanomold to form one-dimensional (1D) NWs using different filling techniques. The electrochemical deposition (ED) [27], chemical vapor deposition (CVD) [28], and atomic layer deposition (ALD) [29] are vastly used in a growth process because of their ability to generate large-scale consistently ordered arrays of 1D NWs. Conversely, these processes have certain limiting factors; when the aspect ratio of the AAO channels increases, it creates porosity and critical stress issues.

In the vacuum die casting approach (VDC), the nanowires were formed directly from the alloy melt. The mass transport and kinetics of the molten metal filling in the high aspect ratio nanochannels with substantial time, reduces the porosity and assist in attaining defect-free NWs. The geometry of the NWs in the VDC process was controlled by the AAO nanochannels dimension. The AAO template-assisted VDC approach performs admirably in synthesizing 1D NWs with exceptional purity and homogeneity. The high pressure and temperature utilized in the VDC approach can intensely control material diffusion on the AAO channel, which directly impacts porous deterioration and defect-free NW growth [30]. On the other hand, the VDC approach has been understudied and lacks solid empirical proof for parametric control in NWs tailoring. Henceforth, the VDC-assisted AAO template method requires a detailed investigation of functional parameters in the NWs fabrication.

This study used the AAO template-assisted VDC approach to fabricate indium antimonite (InSb) NWs. Since the InSb is a high potential thermoelectrical material in group III-IV semiconductors with a narrow bandgap (0.17 eV at 300 K) and high thermal conductivity (17 Wm^−1^K^−1^) [31,32]. InSb NWs demonstrate an excellent electron mobility of 2.5 × 104 cm^2^V^−1^s^−1^ and display strong spin-orbit couplings [33,34,35,36]. It is noteworthy to mention that there has been no investigation of InSb NWs fabricated using the VDC technique as per our knowledge. The AAO template was constructed using two different electrolytes (oxalic and phosphoric acid), and it is further assisted as a nanomold in the VDC process to fabricate InSb NWs. The obtained InSb NWs were utilized to fabricate microdevices for determining thermoelectrical performance.

## 2. Experimental Procedure

*AAO-template Synthesis:* The conventional 2-step anodization process has been utilized for the template synthesis on a commercial grade, high-purity aluminum foil (99.7%) of dimension 50 mm × 3 mm [37]. Prior to the anodization treatment, the Al substrate was pre-processed through ultrasonic degreasing and electro-polishing to remove impurities and relieve stress in the substrate. In a simple anodization bath with an Al substrate as anode and an Al sheet as cathode, the first electrochemical anodization process was carried out with the anodization parameters shown in Table S1. The oxide layer generated on the Al substrate due to anodization is removed by immersing in 1.8 wt.% chromic acid (CrO_3_) and 6 vol.% phosphoric acid (H_3_PO_4_) solution for 1 h at 60 °C. The second anodization was conducted using the same parameters as the first, generating a well-defined nanopore AAO structure. Further, the AAO template allowed for immersion in 5 wt.% of phosphoric solution for pore widening, followed by treatment in an aqueous solution containing 5 vol.% hydrochloric acids (HCl) and 8 wt.% cupric chloride (CuCl_2_) to remove the residual Al present in the template.

*InSb bulk fabrication:* The InSb bulk was fabricated by annealing the In and Sb raw materials through the vacuum sealing process. The homogeneously mixed In (4N) and Sb (5 N) were taken in quartz ingot sealed with oxyhydrogen in a vacuum (5.00 × 10^−5^ torr) and heated above its melting point of 700 °C in the furnace for 7 h. Then, it was allowed to cool down for 3 h to obtain InSb.

**Figure 1:**
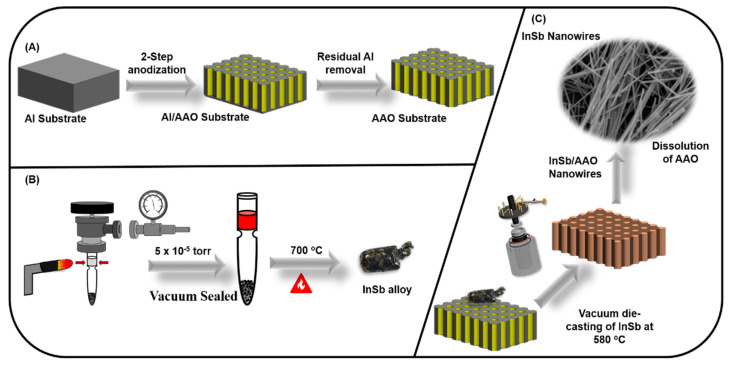
Schematical representation for the fabrication of AAO template (**A**), InSb alloy (**B**), and vacuum die casting process (**C**).

*InSb nanowire fabrication*: The InSb NWs were fabricated using an AAO template-assisted vacuum die-casting process. In this process, the as-synthesized InSb bulk was placed over the AAO template in the vacuum chamber and the working temperature of the chamber was increased to 580 °C for 40 min. Then, the system was allowed to retain room temperature (26 °C) to form the InSb/AAO template. The Insb NWs were extruded from the AAO template by chemical etching (InSb/AAO soaked in 20 vol.% of H_3_PO_4_ for 24 h at 26 °C) process. The obtained InSb suspension was centrifuged and cleaned in DI water to acquire well-defined NWs. Figure 1 illustrates the overall schematic representation of the InSb NWs fabrication process.

*Fabrication of InSb nanowire device:* The InSb NWs obtained from the VDC-assisted template method were dispersed in low concentrations of ethanol and sonicated. The InSb suspension formed was transferred into the Au-patterned silicon substrate for the interconnection of Pt electrodes. FIB was selectively used to deposit Pt–metal to bind the InSb NWs with the Au electrode in assistance with the trimethyl-zyl-cyclopentadienyl-platinum (CH3) 1Pt (CpCH3) injector. The SiO_2_ substrate was pre-processed with an ultrasonicator and cleaned using ethanol/DI water before deposition. Finally, the fabricated single NW devices were utilized to obtain the thermoelectric properties of InSb NW in a four-electrode system.

### 2.1. Characterization

The morphological analysis of as-synthesized materials was evaluated by FESEM, JSM-6900F (field-emission scanning electron microscope, JEOL Ltd., Tokyo, Japan). The structural analysis of InSb nanowires was inspected by HRTEM, JEM-2010 (high-resolution transmission electron microscopy, JEOL Ltd., Tokyo, Japan). The phase and microstructural characteristics of the InSb NWs were examined using D2 PHASER X-ray diffraction (XRD, Bruker, Billerica, MA, USA). The InSb NWs bonding nature was confirmed using a Raman spectroscopy (iHR550, Horiba, Ltd., Kyoto, Japan) instrument. The single NW devices were fabricated with the assistance of the FEI Versa 3D HR-Dual Beam instrument (Thermo Fisher Scientific, Waltham, MA, USA). The thermoelectric properties of the InSb nanowires were measured using a four-electrode setup.

## 3. Results and Discussion

### 3.1. Morphological and Microstructural Analysis

**Figure 2:**
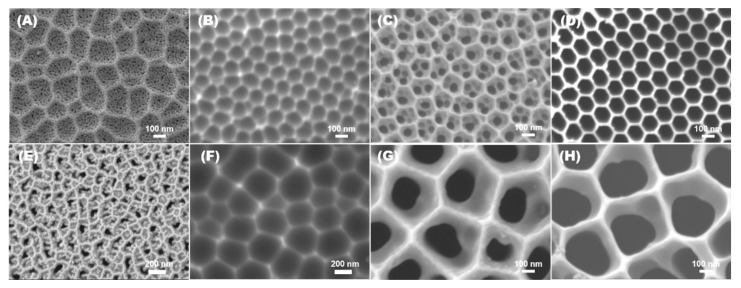
Systematic morphological variation of the AAO template fabricated on oxalic (**A**–**D**) and phosphoric electrolyte (**E**–**H**). FE-SEM image after first-step anodization (**A**,**E**), oxide layer removal (**B**,**F**), second-step anodization (**C**,**G**), and pore widening (**D**,**H**).

The electrochemical oxidation of Al substrate is firmly dependent on the electrolyte used and the process parameters (anodization time and temperature). The diffusion of ionic species between the Al substrate and electrolyte interface determines the oxide layer growth, morphology, size, pore ordering, and plausible mechanism. Figure 2 shows the top view of the AAO template fabricated in the oxalic electrolyte (O-AAO) (A, B, C, & D) and phosphoric electrolyte (P-AAO) (E, F, G, & H), which reveals that the electrolyte has a predominant influence in the pore ordering. The 2-step anodization process was utilized for the ordered aluminum oxide growth of the template. In the sequential approach, the pore arrangements evolve in each phase regardless of oxalic and phosphoric electrolytes, as shown in FE-SEM images (Figure 2), after first-step anodization (A and D), oxide layer removal (B and F), second-step anodization (C and G), and the pore widened process (D and H). The ionic diffusion of the electrolyte towards the anode is attributed to the definite arrangement of pores in the AAO template, which is evident from the FE-SEM images.

**Figure 3:**
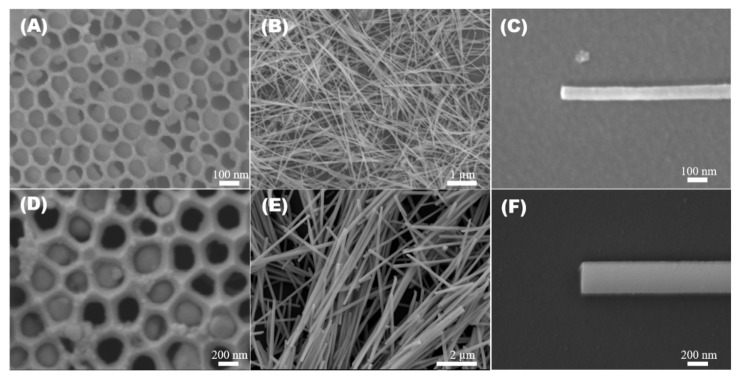
FE-SEM images of InSb embedded over the oxalic and phosphoric AAO template using the vacuum die casting process (**A**,**D**), InSb nanowires synthesized using the oxalic AAO template (**B**,**C**) and InSb nanowires synthesized using the phosphoric AAO template (**E**,**F**).

The fabricated O-AAO and P-AAO templates were further utilized as nanomold in the vacuum die casting method (VDC) to synthesize InSb NWs. The high pressure and temperature in the VDC process melt the InSb compound, allowing it to flow uniformly over the high aspect ratio AAO nanochannels. In the chamber, the temperature was maintained higher than the melting point of InSb at 580 °C for 40 min, which favors the homogeneous diffusion of molten InSb into the AAO templates. Figure 3A,D depicts the InSb alloy embedded over the O-AAO and P-AAO template. The InSb NWs formed were extracted by the dissolution of the AAO template in 20% vol. of H_3_PO_4,_ which merely reacts with aluminum oxide and has no effect on InSb NWs. Figure 3B,E indicates the InSb NWs extracted from O-AAO and P-AAO templates. The EDX spectrum in Figure S1A,B reveals that the elemental composition of synthesized NWs is merely made of In and Sb with a composition ratio of approximately about 1:1. The elemental mapping of InSb NW in Figure S2 indicates an homogenous distribution of the In and Sb element.

**Figure 4:**
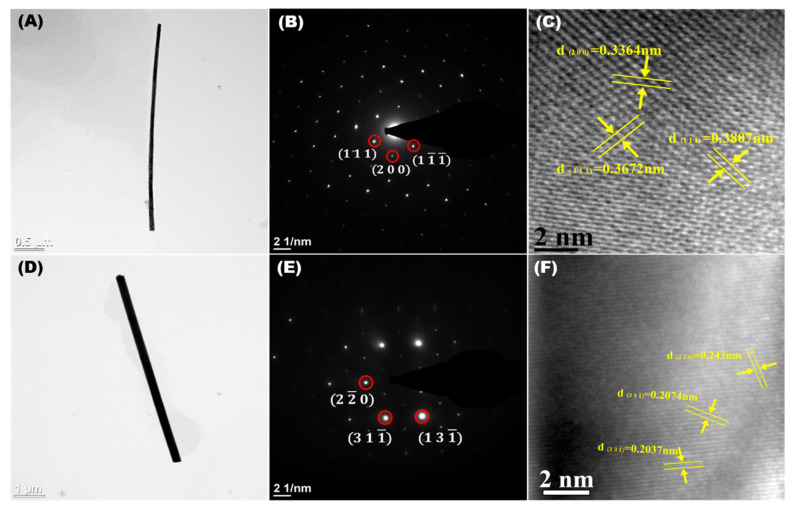
TEM image (**A**), selected area diffraction (**B**), and the HR-TEM (**C**) of a single InSb nanowire fabricated using O-AAO. TEM image (**D**), selected area diffraction (**E**), and the HR-TEM (**F**) of a single InSb nanowire fabricated using P-AAO.

The morphology and crystal structure of synthesized InSb NWs were examined by TEM. Figure 4A,D depicts the typical TEM images of single InSb O-NW and InSb P-NW. The corresponding SAED pattern marked in Figure 4B,E reveals the polycrystalline nature of the InSb O-NW and InSb P-NW. The obtained lattice fringes of 0.33 and 0.36 nm correspond to the (200) and (111) planes for InSb O-NW and 0.24 and 0.20 nm oriented in (220) and (311) planes for P-NW explicit the zinc-blende crystal phase [30,31]. The obtained results confirm that the growth of InSb NWs was preferentially oriented along the zone axis (Figure 4C,F).

**Figure 5:**
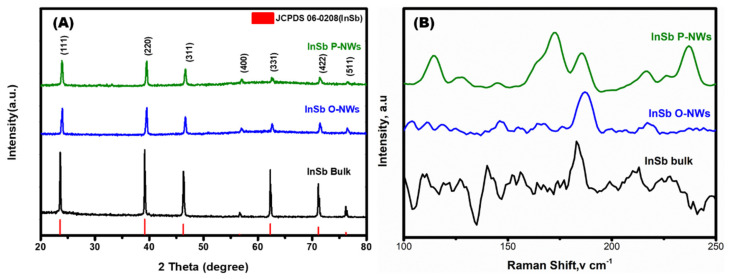
(**A**) XRD and (**B**) Raman pattern obtained for InSb bulk, Insb O-NWs, and InSb P-NWs.

The crystalline structure and characteristics of the Insb NWs synthesized using the AAO template-assisted VDC process were scrutinized using X-ray diffraction analysis. Figure 5A shows the XRD spectra obtained for InSb bulk, InSb O-NWs, and InSb P-NWs. The peaks observed approximately at 24.01°, 39.51°, 46.68°, 56.8°, 62.63°, 71.31°, and 76.41° are attributed to the miller indices of (111), (220), (311), (400), (331), (422), and (511), respectively. The obtained peaks are in good agreement with JCPDS Card no. 06-0208, which elucidates the zinc-blend crystal structure of InSb. Further, Raman spectroscopy was utilized to understand the bonding nature of the as-prepared materials shown in Figure 5B. Two distinct peaks appear at 172 and 186 cm^−1^ for InSb P-NWs, which indicates the transverse-optical (TO) and longitudinal-optical (LO) phonon modes, respectively [28]. However, the InSb O-NWs show an apparent peak at 186 cm^−1^ LO, indicating phonon mode. The distinct splitting of the phonon peaks implies that the InSb P-NW is firmly crystalline compared to InSb O-NWs. Thus, the SEM, HRTEM, XRD, and Raman analysis confirm the successful formation of InSb NWs via the AAO template-assisted VDC method. The InSb P-NW exhibits a higher structural uniformity across the length and crystallinity than InSb O-NWs. Hence, the InSb P-NWs were further utilized for single nanowire device fabrication to determine its thermoelectrical properties.

### 3.2. Thermoelectrical Property of InSb NWs

**Figure 6:**
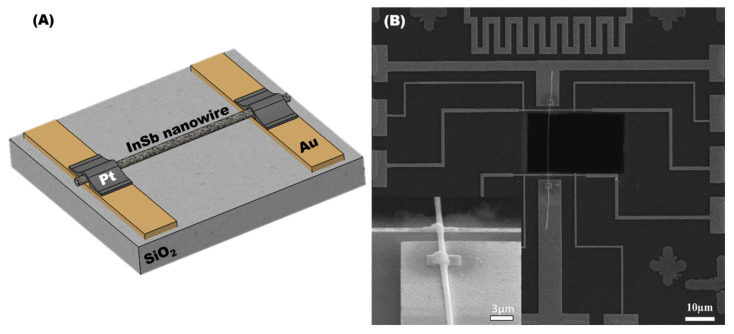
(**A**) Schematic representation of InSb P-NW device fabrication; (**B**) the SEM image of the InSb P-NW connected with a Pt electrode on Au-patterned Si substrate, with a magnified image in the inset.

**Figure 7:**
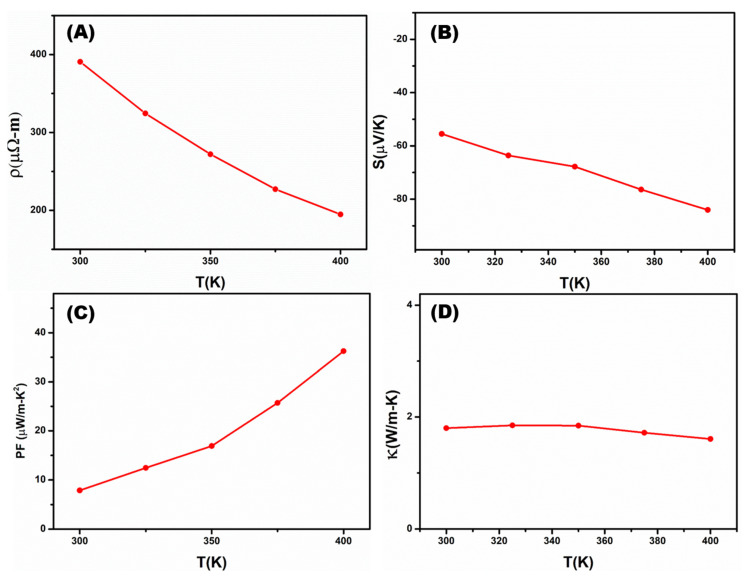
Resistivity (**A**), Seebeck coefficient **(B**), power factor (**C**), and thermal conductivity (**D**) of InSb P-NWs as a function of temperature.

The single NW devices were utilized to evaluate the essential thermoelectrical properties of the InSb P-NWs. The InSb P-NW were integrated onto the patterned silicon substrate through Pt electrode with the assistance of FIB, and the systematic procedure was illustrated in Section 2.1. Figure 6A,B shows a schematic representation of the InSb P-NW device and the corresponding microscopic pictures. The single NW device was used for determining the electrical resistivity (ρ) and Seebeck coefficient (S) via four-point measurements. All the experiments were measured as a function of temperature in the range from 300 to 400 K. In the four-point probe technique, electrodes 1 and 2 applied an alternating current to the NW, and electrodes 3 and 4 measured the RMS value of the voltage. From R = V/I, the resistance value of the sample was obtained. Then ρ was evaluated from ρ = RA/L, where A indicates the cross-sectional area, and L is the length of nanowires determined by SEM after electrical characterization. Figure 7A shows the measured electrical resistivity of the InSb P-NW device. The magnitude of the resistivity decreased with increasing temperature, which illustrates the electrical behavior of the semiconductor. The resistivity of the InSb NW was measured to be 390.6 µΩ-m at ambient temperature.

For the Seebeck coefficient (S) measurement, the characterization was based on the electrodes voltage and temperature difference. Two thermometers were placed at the electrodes to define the temperature difference shown in Figure S3. In order to generate the temperature gradient, a heater with a frequency 1ω and a magnitude equal to I•Sin(ωt) was applied at one end of the NW. The applied heat (Q) was directly proportional to the I^2^•sin^2^(ωt)•R, where R represents the electrical resistance of the NW. As sin^2^α = [(1 − cos2α)/2] indicates, the frequency of 2ω was utilized to heat the heater [38]. By measuring the temperature difference as well as the associated voltage on the electrodes, S can be calculated from S = (△V)/(△T). The Seebeck coefficient of the InSb NW was marked in Figure 7B, consistent with the electrical resistivity behaviour. It is presumed that the negative trend is responsible for n-type electrical transport characteristics, owing to the higher mobility of electrons than holes. The Seebeck value for the n-type InSb P-NW at room temperature (300 K) was −55.5 µV/K, which indicates that the Femi level lay well inside the conduction band. Figure 7C describes the evaluated power factor (PF) of InSb P-NW. The temperature-dependent PF is linearly proportional to the Seebeck coefficient and calculated to be 7.9 µW/m-K^2^ at room temperature (300 k), ascribing enhanced electronic transport properties.

The thermal conductivity was measured using the self-heating 3ω method, which relies on the suspended conductive NW (Figure 6B). When AC current I•Sin(ωt) was applied to the sample, it generated temperature variation at a frequency of 2ω and induced a third harmonic voltage V_3ω_ in the voltage contact due to resistance at the corresponding temperature. The 3ω method can be expressed as [38]:

(1) $$V_{3\omega }=\frac{4I_{0}^{3}\;{\mathrm{LRR}}^{\prime }\;}{{\;\pi }^{4\;}\;\kappa S\surd (1+{\left(2\omega \gamma \right)}^{2}}$$

where I and ω indicate the amplitude and frequency of the alternating current applied on the NW, respectively; R and R’ denote the resistance and derivative of resistance at the corresponding temperature, respectively; κ is the thermal conductivity; S is the NW cross-section area; and γ is the characteristic thermal time constant. Figure 7D shows the thermal conductivity plot, and the κ is found to be 1.63 W/m-K at 400 K temperature.

**Figure 8:**
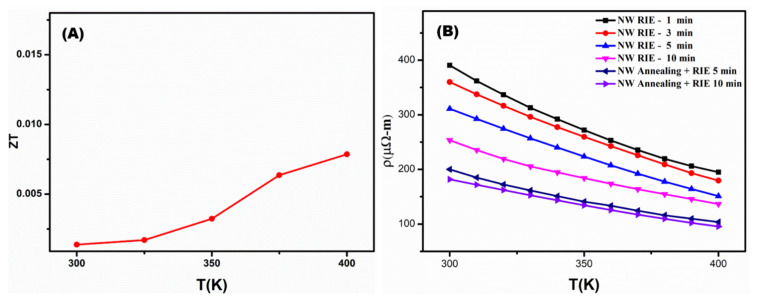
(**A**) ZT-obtained InSb P-NW as a function of temperature. (**B**) The resistivity of InSb P-NWs was measured for different RIE times and annealing.

The figure of merits (ZT) was shown in Figure 8A as a function of temperature. The dimensionless value of ZT increases with increasing temperature. The thermoelectrical performance obtained for the InSb NWs is low compared to the reported values [31,32]. When the NWs were synthesized utilizing the AAO template assisted-VDC approach, a thin oxide layer was formed in the NWs (Figure S4). As a consequence, the oxide layer causes a non-ohmic contact with the Pt electrode deposited on the NW (Figure 6) used for the thermoelectric measurements, which deteriorates the electron transportation, initiating high resistivity. In order to suppress these effects, the reactive-ion etching (RIE) and annealing treatment was carried out on InSb P-NWs. The curves in Figure 8 elucidate that the increase in the RIE treatment time significantly decreases the electrical resistivity. The linear variation of RIE treatment time towards electrical resistivity decreases from 390.6 to 253.5 µΩ-m. In conjunction with annealing the InSb NW at 350 °C for 3 h, the RIE process significantly reduces the electrical resistivity. The annealed InSb sample with a 10 min RIE treatment time yields the lowest resistivity of 181.6 µΩ-m. Given the fact that the defects on the fabricated InSb P-NWs can be optimized to obtain enhanced thermoelectrical performance through the VDC method. This potential VDC manufacturing approach opens up new possibilities in NW synthesis for use in practical thermoelectrical applications.

## 4. Conclusions

The one-dimensional InSb nanowires were synthesized from the AAO template-assisted vacuum die-casting process. The high pressure and temperature in the vacuum die-casting method influenced the homogenous diffusion of InSb into the high aspect ratio AAO template. According to TEM, FE-SEM, Raman, and X-ray diffraction analyses, the InSb nanowires obtained from the dissolution process show homogeneity and crystallinity. The thermoelectric properties of a single InSb nanowire device fabricated by FIB are determined. Using the reactive-ion etching technique in conjunction with the annealing treatment, the surface-modified nanowire improved the thermoelectric performance remarkably. The results demonstrate excellent fabrication of InSb nanowires to be used in thermoelectric applications.

## Data Availability

The data presented in this study are available on request from the corresponding author.

## Supplementary Materials

The following supporting information can be downloaded at: https://www.mdpi.com/article/10.3390/nano12122032/s1, Table S1: Anodization parameters used for the synthesis of AAO template; Figure S1: A,B EDX observed the elemental composition of synthesized NWs obtained for (A) InSb O-NWs and (B) InSb P-NWs; Figure S2: Elemental mapping of the InSb nanowires, showing In (A) and Sb (B); Figure S3: InSb P-NW setup for Seebeack effect measurement (A) temperature and (B) Voltage difference; Figure S4: TEM image of single InSb P-NW with thin oxide layer.
