# Supplementary material for: Tailoring InSb Nanowires for High Thermoelectric Performance Using AAO Template-Assisted Die Casting Process
Source: Nanomaterials (Basel). 2022 Jun 13;12(12):2032. doi: 10.3390/nano12122032 (PMC9227088; doi:10.3390/nano12122032)
Supplement: Supplementary file 1 [file nanomaterials-12-02032-s001.zip › nanomaterials-1731300-supplementary.pdf]

nanomaterials

Supplementary materials

Tailoring InSb Nanowires for High Thermoelectric Performance Using AAO Template-Assisted Die Casting Process

Alangadu Kothandan Vivekanandan [1], Chen-Wei Lee [1], Rui-Zhe Wu [1], Wei-Han Tsai [2], Shih-Hsun Chen [1,*], Yang-Yuan Chen [2,*] and Chia-Ting Lin [3]

1 Department of Mechanical Engineering, National Taiwan University of Science and Technology, Taipei 10607, Taiwan; vivekak77@gmail.com (A.K.V.); chenweibear1007@gmail.com (C.-W.L.); jerrywu4416@gmail.com (R.-Z.W.)
2 Institute of Physics, Academia Sinica, Taipei 11529, Taiwan; ham0444@gmail.com
3 Engineer, Chemical systems Research Division, National Chung-Shan Institute of Science and Technology, Taoyuan 325, Taiwan; w409635@gmail.com (C.-T.L.)
* Correspondence: shchen@mail.ntust.edu.tw (S.-H.C.); cheny2@gate.sinica.edu.tw (Y.-Y.C.)

Table S1. Anodization parameters used for the synthesis of AAO template.

Electrolyte	Potential (V)	Electrolyte temperature (° C)	Anodization time (h)		Pore widening (min)
			First anodization	Second anodization	
3 wt.% $H_2C_2O_4$	40	5	1	48	30
1 vol.% H_3PO_4	150	5	1	72	20

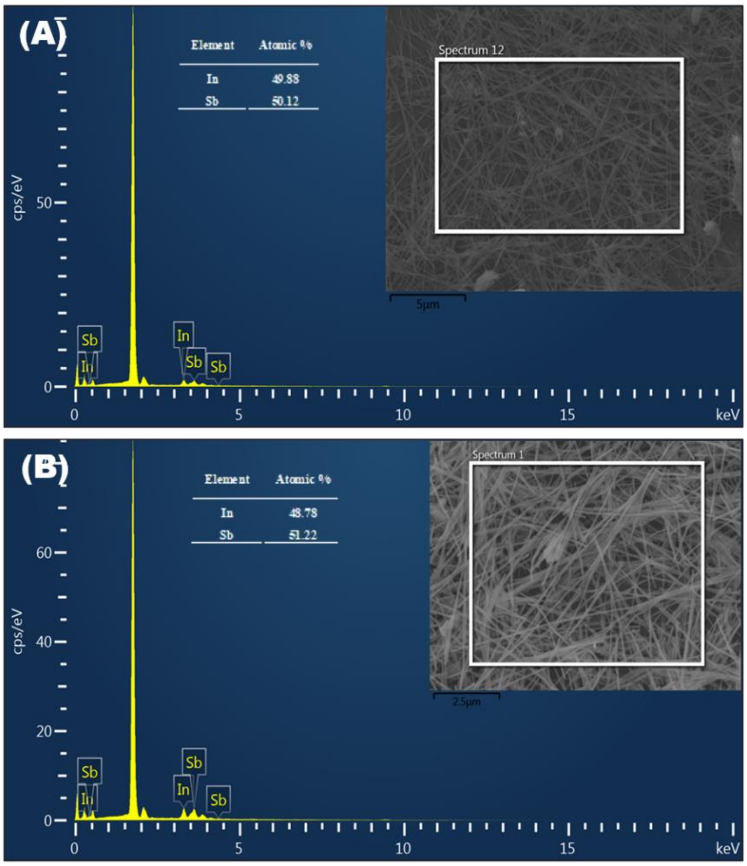

Figure S1. (A, B) EDX observed elemental composition of synthesized NWs obtained for (**A**) InSb O-NWs and (**B**) InSb P-NWs.

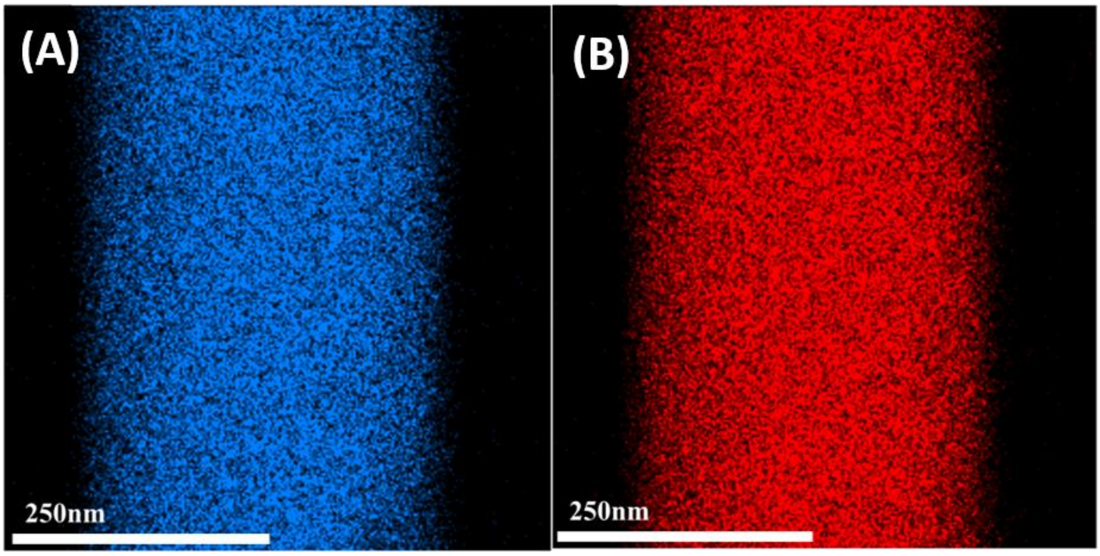

Figure S2. Elemental mapping of the InSb nanowires, showing In (**A**), and Sb (**B**).

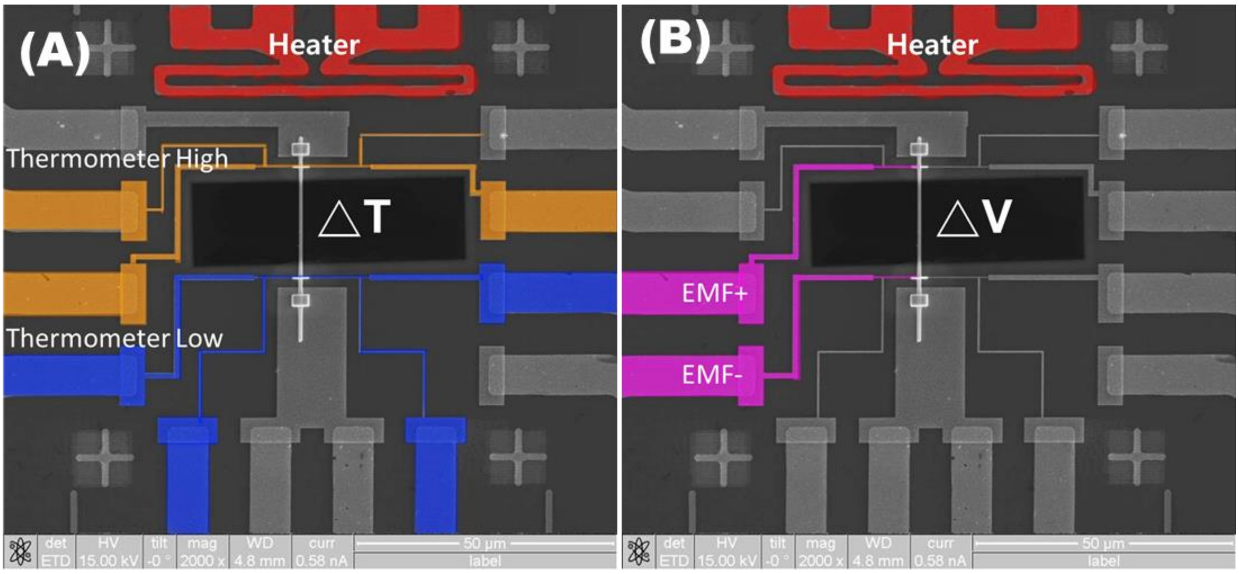

Figure S3. InSb P-NW setup for Seebeack effect measurement (**A**) temperature and (**B**) Voltage difference.

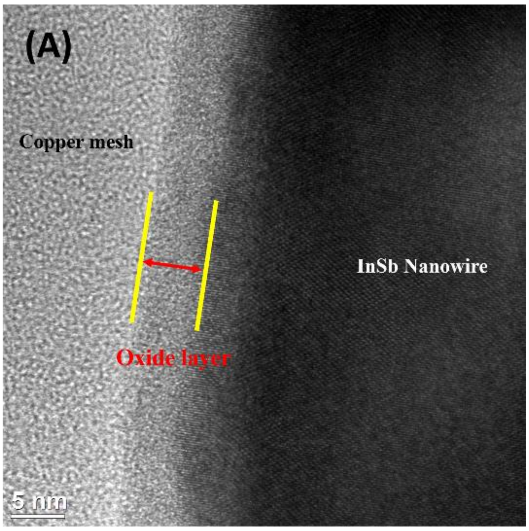

Figure S4. TEM image of single InSb P-NW with thin oxide layer.
